# Marine Mammal *Brucella* Genotype Associated with Zoonotic Infection

**DOI:** 10.3201/eid1403.070829

**Published:** 2008-03

**Authors:** Adrian M. Whatmore, Claire Dawson, Pauline Groussaud, Mark S. Koylass, Amanda King, Stephen J. Shankster, Annette H. Sohn, Will S. Probert, Wendy L. McDonald

**Affiliations:** * Veterinary Laboratories Agency, Addlestone, UK; † University of California, San Francisco, California, USA; ‡ California Department of Public Health, Richmond, California, USA; § Ministry of Agriculture and Forestry, Wallaceville, New Zealand

**Keywords:** Brucella, marine mammal, genotyping, letter

## Abstract

Marine Mammal Brucella Genotype Associated with Zoonotic Infection

**To the Editor:** Brucellosis is a zoonotic disease that remains endemic to many parts of the world. There are 6 classic *Brucella* species described with different preferred hosts. Human disease is most commonly associated with consumption of unpasteurized dairy products or with occupational exposure for veterinarians, agricultural workers, laboratory workers, meat industry workers, and hunters.

In recent years, it has become clear that novel members of the genus, yet to be formally named, are associated with a variety of marine mammal species, particularly dolphins, porpoises, and seals (*1*). To date there are 3 reports in the literature of naturally acquired infection of humans with *Brucella* species originating from marine mammals (*2**,**3*) One other case, representing infection of a laboratory worker, has also been reported (*4*). Two of the naturally acquired cases were reported in Peru (*2*). One person had consumed raw shellfish and swam in the Pacific Ocean but did not report any direct contact with marine mammals; the second person reported infrequent visits to the coast and no contact with marine mammals but had consumed raw shellfish. An additional naturally acquired case was recently reported from New Zealand, where extensive molecular testing characterized the strain involved as a marine mammal type (*3*). This patient again reported no exposure to marine mammals but did report that he fished regularly, had contact with uncooked fish bait, and consumed raw snapper. The cases in Peru were notable for severe, atypical symptoms; both patients had symptoms of neurobrucellosis. The New Zealand case was associated with spinal osteomyelitis (*3*). In contrast, the laboratory-acquired infection was mild and uncomplicated (*4*).

We have characterized these isolates by a variety of molecular approaches in conjunction with ongoing studies, which examine genetic diversity within *Brucella* species isolated from marine mammals. Multilocus sequence analysis (*5*) showed that all 3 isolates from naturally acquired human infection with *Brucella* species from marine mammals shared an identical genotype (ST27). In previous characterization of 56 *Brucella* isolates from marine mammals, ST27 was found only once. Strain F5/99, originally isolated from an aborted bottlenose dolphin fetus off the western coast of the United States (*6*), shares this genotype. Use of an alternative typing approach, based on restriction fragment length polymorphism analysis of outer membrane protein–encoding genes (*7*), gave identical findings. Again, all 3 isolates derived from naturally acquired human infection represent an identical genotype. This genotype is shared only by strain F5/99 among a collection of 120 *Brucella* isolates from marine mammals characterized by this method. Finally, isolates were characterized by a variable number of tandem repeats–based typing approach (*8*). When comparing profiles at 6 loci with a relatively slow evolutionary speed, previously shown to be useful for dividing *Brucella* isolates into species groups (*8*), we determined that F5/99 and the 3 naturally acquired human isolates share a unique profile not seen in any of >1,400 isolates of marine or terrestrial *Brucella* species examined to date. In contrast, the strain associated with laboratory-acquired infection (*4*) was not a member of ST27 but belonged to ST23, a genotype that is predominantly associated with porpoises (*9*).

It is clear from these findings that the 3 cases of naturally acquired infection with *Brucella* species originating from marine mammals reported to date were caused by closely related organisms. The particular genotype concerned, ST27, is rare in our collection, having been noted only once in marine mammals. However, this may reflect the fact that most isolates examined to date originated from northern Europe; only 5 isolates in our collection originated from Pacific waters. It is possible that isolates of this genotype are predominantly or exclusively associated with regions other than those extensively sampled to date. Indeed, examination of the literature provides further evidence for the presence of this genotype in marine mammals in the Pacific. BLAST (www.ncbi.nlm.nih.gov/blast) comparison of outer membrane protein sequences from a minke whale isolate originating in the North Pacific (*10*) showed a close match with the equivalent sequence from F5/99.

Although numbers are currently small, the isolation of an identical genotype from all 3 cases of naturally acquired human infection derived from marine mammal *Brucella* species raises the possibility of increased zoonotic potential associated with this genotype. Furthermore, where diagnosis is based on serologic testing alone, it is possible that human infection with marine mammal *Brucella* species may go unnoticed. Members of ST27 may be more pathogenic to man, per se. Alternatively, they may be associated with natural hosts or circulate through intermediaries that make contact with humans more likely. Notably, none of the 3 patients reported direct contact with marine mammals, though all had consumed raw seafood. These findings clearly suggest that more extensive studies of the presence and distribution of marine mammal *Brucella* genotypes, particularly ST27, in waters other than those of northern Europe would be valuable for clarifying the natural habitat of ST27. Furthermore, relevant authorities should be aware of the potential for zoonotic disease caused by this *Brucella* genotype particularly, but not exclusively, where occupation or lifestyle may make exposure more likely.

Brucellosis research at the Veterinary Laboratories Agency is supported by the UK Department of Environment, Food and Rural Affairs (Defra).
